# On the origin of the *P*-element invasion in *Drosophila simulans*

**DOI:** 10.1186/s13100-025-00345-0

**Published:** 2025-02-26

**Authors:** Filip Wierzbicki, Riccardo Pianezza, Divya Selvaraju, Madeleine Maria Eller, Robert Kofler

**Affiliations:** 1 Institut für Populationsgenetik, Vetmeduni Vienna, Vienna, Austria; 2 Vienna Graduate School of Population Genetics, Vienna, Austria

## Abstract

The horizontal transfer (HT) of the *P*-element is one of the best documented cases of the HT of a transposable element. The *P*-element invaded natural *D. melanogaster* populations between 1950 and 1980 following its HT from *Drosophila willistoni*, a species endemic to South and Central America. Subsequently, it spread in *D. simulans* populations between 2006 and 2014, following a HT from *D. melanogaster*. The geographic region where the spread into *D. simulans* occurred is unclear, as both involved species are cosmopolitan. The *P*-element differs between these two species by a single base substitution at site 2040, where *D. melanogaster* carries a ‘G’ and *D. simulans* carries an ‘A’. It has been hypothesized that this base substitution was a necessary adaptation that enabled the spread of the *P*-element in *D. simulans*, potentially explaining the 30-50-year lag between the invasions of *D. melanogaster* and *D. simulans*. To test this hypothesis, we monitored the invasion dynamics of *P*-elements with both alleles in experimental populations of *D. melanogaster* and *D. simulans*. Our results indicate that the allele at site 2040 has a minimal impact on the invasion dynamics of the *P*-element and, therefore, was not necessary for the invasion of *D. simulans*. However, we found that the host species significantly influenced the invasion dynamics, with higher *P*-element copy numbers accumulating in *D. melanogaster* than in *D. simulans*. Finally, based on SNPs segregating in natural *D. melanogaster* populations, we suggest that the horizontal transfer of the *P*-element from *D. melanogaster* to *D. simulans* likely occurred around Tasmania.

**Supplementary Information:**

The online version contains supplementary material available at 10.1186/s13100-025-00345-0.

## Introduction

**Fig. 1:**
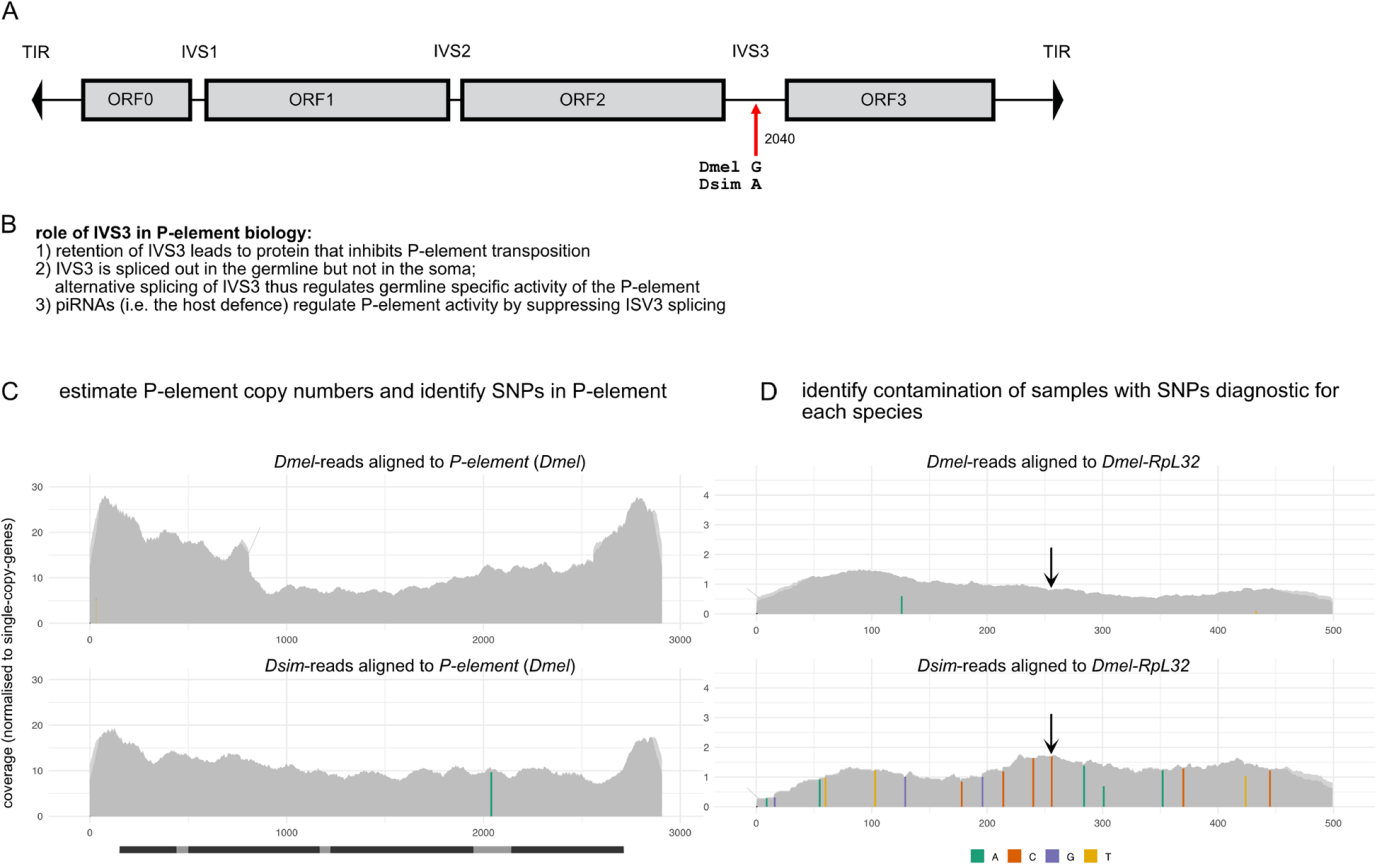
**A** Overview of the *P*-element. The ORFs, introns (IVS1-3), TIRs (black triangles), position of the base substitution at position 2040 in intron 3 (IVS3; red arrow) and the major allele of each species are shown. **B** Summary of the role of IVS3 in *P*-element biology [29, 49, 59]. **C** Our tool, DeviaTE, enables us to estimate *P*-element copy numbers and identify SNPs in the *P*-element. Reads are aligned to the *P*-element and single-copy-genes (the corresponding orthologous for each species). *P*-element copy numbers are estimated as coverage of the *P*-element normalized by the coverage of single-copy genes. In this example, reads from *D. simulans* and *D. melanogaster* are aligned to the *P*-element. The frequency of alternative alleles are shown as colored bars. Note, that the *P*-element from *D. simulans* has a different allele (A: green) at position 2040. **D** Diagnostic SNPs enable us to identify contamination of data with reads from a different species. For example, contamination of a *D. melanogaster* sample with *D. simulans* reads can be identified based on SNPs that are fixed for alternative alleles in the two species (e.g. arrow)

Transposable elements (TEs) are short DNA sequences that selfishly spread in genomes [7], found in almost all eukaryotic species investigated so far [67]. Although some TE insertions may be beneficial to the host, it is thought that most TE insertions are deleterious [9, 35, 42]. Therefore, to curb the activity of TEs, hosts have developed sophisticated defense mechanisms, frequently involving small RNAs [4, 51]. Once TEs are silenced by the host, TE insertions will gradually accumulate mutations that may erode their ability to multiply in genomes [3]. To escape this gradual decay, a TE may occasionally horizontally transfer (HT) to a novel host species. This HT could trigger an invasion, where the TE multiplies in the novel species, until it is again silenced by the host’s defense. Based on indirect evidence, such as the sequence similarity of TEs among species, it was suggested that HT is highly abundant among insect species [43]. There are however also a few cases with more direct evidence for a HT, e.g. when a TE is absent in earlier collected strains but present in later ones [1, 24, 45, 52, 54]. One of the best documented cases is the HT of the *P*-element in *D. melanogaster* [1, 14, 22]. The *P*-element is one of the most widely studied eukaryotic TEs. It is a DNA transposon, with a size of 2907bp and 4 exons [36] (Fig. 1A). It has been used as a versatile tool in molecular biology, for example in mutagenesis screens, enhancer traps and as vector for transforming multicellular organism [12, 41, 50]. Investigations of natural *D. melanogaster* populations show that the *P*-element is absent in all strains collected around 1950, whereas it is present in most strains collected around 1980 [1]. Therefore, it was predicted that the *P*-element rapidly invaded natural *D. melanogaster* populations between 1950 and 1980 [1, 22]. It was further shown that the *P*-element invasion in *D. melanogaster* was likely triggered by a HT from *D. willistoni*, a species endemic in Central and South America [6, 13]. As *D. melanogaster* colonized the Americas within the last two centuries, this habitat expansion was a crucial prerequisite for the HT of the *P*-element.

Early studies showed that the *P*-element did not initially spread to related species, including the closely related *D. simulans* [5, 13, 15]. *D. simulans* and *D. melanogaster* diverged about 2–3 mya and both species are human commensals with a world-wide distribution [8, 18, 27, 32, 60]. However, when we and colleagues in previous works investigated *D. simulans* strains collected over the last few decades, we realized that the *P*-element was rare in strains collected around 2006 but abundant in strains collected around 2014 [19, 24]. Presence of the *P*-element in *D. simulans* was later confirmed for populations in Japan and Brasil [39, 68]. The donor species of the HT is likely *D. melanogaster*, as the *P*-element between *D. melanogaster* and *D. simulans* differs just by a single base at site 2040 (*D. melanogaster* ‘G’, *D. simulans* ‘A’; [24]). For the HT between *D. melanogaster* and *D. simulans*, the geographic region where the HT occurred cannot be inferred from their habitats, as both species are largely cosmopolitan [8] However, the data indicate that the *P*-element invaded natural *D. simulans* populations around 30 - 50 years after the invasion of natural *D. melanogaster* populations. What could be the reason for this lag time? Two possible hypothesis have been suggested [24]. First, that HT could be very rare, and thus the observed lag time may simply be the waiting time for the rare HT event. Second, the single base substitution at site 2040 ($G->A$) of the *P*-element was a necessary adaptation for the spread of the *P*-element in *D. simulans*. Hence, the lag time could be the waiting time for the base substitution plus the HT of the novel variant. The hypothesis that the base substitution at site 2040 was necessary is plausible as it is found in the third intron of the *P*-element (IVS3), which has a central role in its biology. The *P*-element is solely active in the germline but not in the soma. This tissue specific activity is regulated by alternative splicing of IVS3, which is only spliced out in germline [30]. Retention of this intron leads to the translation of proteins that inhibit the transposition of the *P*-element [49]. The small RNA based host defense acts by suppressing splicing of IVS3 in the germline [59] (for summary see Fig. 1B).

To test whether the base substitution at site 2040 $G->A$ was a necessary precondition that enabled the spread of the *P*-element into *D. simulans*, we introduced *P*-elements with both alleles into inbred *D. melanogaster* and *D. simulans* strains via micro-injection. We monitored the ensuing invasions in experimental populations of both species for around 60 generations by sequencing the populations at regular time intervals. Our data show that *P*-elements with the allele 2040G are able to rapidly spread in populations, similarly to *P*-elements with the allele 2040A. We thus conclude that the base-substitution 2040 $G->A$ was not necessary for the invasion of the *P*-element in *D. simulans*. The reason for the lag time between the invasions of *D. melanogaster* and *D. simulans* is therefore likely the scarcity of HT events. Furthermore, based on variation in the *P*-element sequences segregating in *D. melanogaster* populations, we estimate that the HT of the *P*-element from *D. melanogaster* to *D. simulans* most likely happened around Tasmania.

## Results

### Invasion dynamics of *P*-element with 2040A and 2040G

To test whether the base substitution 2040 $G->A$ was a necessary adaptation for the *P*-element invasion in *D. simulans*, we aimed to monitor the invasions of both versions of the *P*-element (2040A and 2040G) in experimental populations of *D. simulans* and *D. melanogaster*. To trigger the invasions, we obtained plasmids carrying the *P*-element with both alleles. We introduced both plasmids (2040G and 2040A) into inbred strains of *D. melanogaster* (Dmel68; collected 1954 in Israel) and *D. simulans* (Dsim001; collected in 1956 in Georgetown). Both strains were collected before the *P*-element invaded natural populations of these two species (*D. melanogaster* $\approx 1950-1980$; *D. simulans* $\approx 2006-2014$ [1, 19, 22]) and are thus free of any *P*-element insertions (Supplementary Fig. S1). To start the experimental populations, we mated 25 transformed flies with 25 naive flies and then mixed them with another 200 naive flies. We kept flies at a population size of $N=250$ at a temperature of 25$^{\circ }$C for $\approx 60$ generations using non-overlapping generations and 3 replicate populations. We measured the status of the invasion by sequencing the populations as pools (Pool-Seq [53]) at about all 10 generations. The abundance of the *P*-element was estimated with our tool DeviaTE [65]. We aligned short-read data to a set of sequences consisting of the *P*-element and three single-copy-genes (*rhi*, *tj*, *RpL32*; we used the corresponding ortholog for each species). Copy numbers were estimated by normalizing the coverage of the *P*-element to the coverage of the single-copy-genes. For example, if the *P*-element has a coverage of 30 and the single copy genes an average coverage of 5, we infer that the sample has roughly 6 *P*-element insertions per haploid genome (Fig. 1C). DeviaTE also provides us with the frequency of each allele in the *P*-element. This enables us to validate whether the correct *P*-element allele is present in a sample (i.e. 2040A or 2040G; Fig. 1C). Our approach has an additional advantage: based on the alignment of the reads to the single copy genes we can identify diagnostic SNPs specific for each of the two species (*D. simulans* and *D. melanogaster*; Fig. 1D; Supplementary Fig. S2; Supplementary data S1, S2). Based on these diagnostic SNPs, we confirmed, for all data, that we analyzed the correct *P*-element allele and the correct species (Supplementary Fig. S3).

**Fig. 2:**
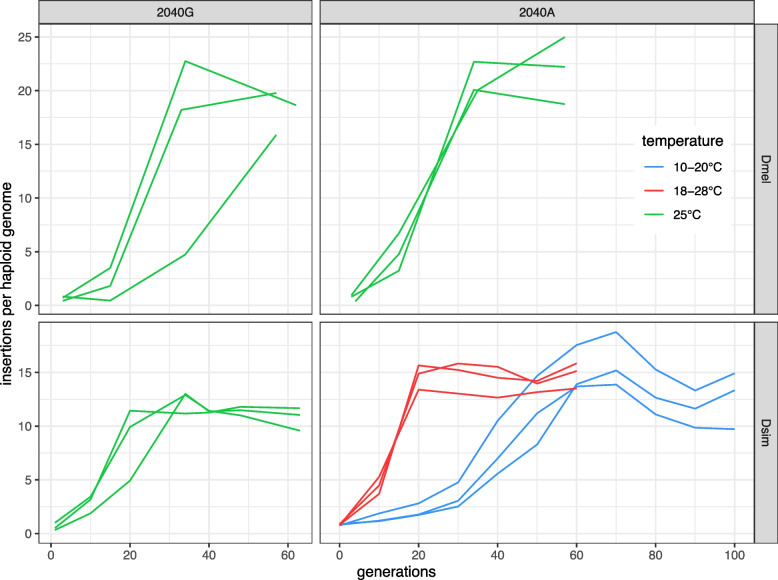
Invasion dynamics of two different *P*-element versions in *D. melanogaster* and *D. simulans*. The *P*-element either has a ‘G’ (i.e. the melanogaster SNP) or an ‘A’ (i.e. the simulans SNP) at position 2040. Note, that for the *P*-element with 2040A in *D. simulans*, we relied on previously published data, where different experimental conditions where used (e.g. temperature; [25, 26])

However, when analyzing the data we realized, belatedly (partly due to COVID), that the invasions of the *P*-element with 2040A failed in *D. simulans* populations (i.e. all *P*-element insertions were lost after a few generations). Ironically, this is the allele of the *P*-element that rapidly spread in natural and experimental *D. simulans* populations [24–26]. In our experience, triggering TE invasions in experimental populations is challenging and frequently fails due to unclear reasons (see Discussion). We substituted publicly available data from our previous work for the missing data [25, 26]. In this previous work, we monitored invasions of the *P*-element with 2040A in experimental *D. simulans* populations. Notably, these invasions were not triggered by micro-injection (as done in this work), but were occurring naturally, as the base population was sampled at the early stages of the *P*-element invasion in *D. simulans* (Florida in 2010). These populations were kept at two different temperatures (hot and cold conditions; where the temperature fluctuated between 10–20$^{\circ }$C and 18–28$^{\circ }$C, respectively). As the activity of the *P*-element depends on temperature [38], we observed a rapid increase in copy numbers in hot conditions and a slow increase in cold conditions [25, 26] (Fig. 2). Additionally, this previous work is based on an outbred population, while inbred strains were used in this work. Finally the population size was larger in the previous work ($N=1250$) than in this work ($N=250$).

We found that the *P*-element with 2040G rapidly spread in all three replicates of *D. simulans* populations (Fig. 2). The invasion of 2040G in *D. simulans* reached a plateau (i.e. the level where *P*-element copy numbers stabilize) around generation 20–30, which is comparable to the invasion of *P*-element with 2040A at hot conditions, where a plateau was also reached around 20 generations (Fig. 2). Furthermore, the level of the plateau are similar between 2040G and 2040A (yet significantly different; average copy number at the latest generation $n_{2040G}=10.76$, $n_{2040A-hot}=14.83$, $n_{2040A-cold}=12.66$; t-test ‘A’ vs ‘G’: $p = 0.029$). Differences in population size can likely not explain the slightly lower plateau level of 2040G, as small populations (2040G) are expected to accumulate more TEs than large ones (2040A), whereas we observed fewer (Supplementary Fig. S4). We thus conclude that the *P*-element with 2040G is able to rapidly spread in *D. simulans* populations. Furthermore, the allele 2040A was not a necessary adaptation that enabled the spread of the *P*-element in *D. simulans*. Overall, it seems that the SNP at position 2040 has very little impact on the invasion dynamics of the *P*-element (Fig. 2). This is also remarkable, given the differences in experimental conditions between the invasions of the *P*-element with 2040G (single inbred line; $N=250$; 25$^{\circ }$C) and 2040A (outbred population; $N=1250$; hot and cold conditions). To further investigate the impact of the SNP at position 2040, we studied the invasion dynamics of the *P*-element with 2040A and 2040G in *D. melanogaster* populations (Fig. 2). The copy numbers of both *P*-element versions increased rapidly in all replicates (Fig. 2). In most replicates the copy numbers reached a plateau at around generation 30, with about 20 copies per haploid genome. Also, in *D. melanogaster*, the copy numbers at the plateau are very similar between both versions of the *P*-element ($n_{2040G-g60}=18.09$, $c_{2040A-g57}=21.98$; t-test ‘A’ vs ‘G’, $p = 0.157$). Hence, the data in *D. melanogaster* support the view that the SNP at position 2040 has little impact on the invasion dynamics of the *P*-element. Interestingly, our data suggest that the species has an impact on the level of the plateau of the *P*-element invasion. In *D. simulans* the *P*-element invasions typically plateau at 10–15 copies per haploid genome, whereas the invasions plateau around 20 copies in *D. melanogaster* (Fig. 2; average copy number at the latest generation $n_{sim}=12.75$, $n_{mel}=20.04$; t-test, $p = 0.001$).

In summary, we conclude that the allele at position 2040 (‘A’ or ‘G’) has little impact on the invasion dynamics of the *P*-element in both *D. melanogaster* and *D. simulans*. The allele 2040A was thus not a necessary adaptation in enabling the spread of the *P*-element in *D. simulans* populations. However, our data suggest that the species has an impact on the plateau level of the *P*-element.

### Geographic origin of the HT of the *P*-element between *D. melanogaster* and *D. simulans*

Previous work showed that 2040A (i.e.: the simulans allele) segregates at a low frequency in natural *D. melanogaster* populations [24]. We hypothesized that the frequency of this allele, and perhaps additional alleles of other SNPs, might vary among populations and that this variation in frequency could be used to identify the geographic region where the HT of the *P*-element from *D. melanogaster* to *D. simulans* most likely happened. The most likely geographic origin is the region where the ‘simulans-alleles’ of the *P*-element have the highest frequency in natural *D. melanogaster* populations. To address this question we first performed a survey of *P*-element SNPs in natural populations of *D. melanogaster* and *D. simulans*. For *D. melanogaster*, we utilized 547 samples (individual strains or pooled populations, data from [10, 17, 28, 47, 48, 54]) from 5 continents and for *D. simulans* we used 37 samples from 4 continents (data from [24, 55, 57, 58, 64]; see Supplementary data S3, S4). Using our diagnostic SNPs, we ensured that each sample is free of contamination from the other species (Supplementary Fig. S5).

**Table 1:** Frequency of different *P*-element alleles in natural populations of *D. melanogaster* and *D. simulans*. The sites are sorted by the most pronounced allele frequency difference between *D. melanogaster* and *D. simulans*. Only SNPs with an average frequency difference of $>0.01$ are shown. For each site in the *P*-element (site) we show the reference base (ref), the segregating alleles (alleles) and the average frequency of the alleles in populations of *D. melanogaster* (Dmel) and *D. simulans* (Dsim)

Site	Ref	Alleles	Dmel	Dsim
2040	G	G/A	0.992/0.008	0.00/1.00
32	A	T/A/G	0.535/0.414/0.051	0.00/1.00/0.00
33	A	A/T	0.978/0.021	1.00/0.00
517	A	A/T	0.979/0.021	1.00/0.00
652	C	C/A	0.989/0.011	1.00/0.00

**Fig. 3:**
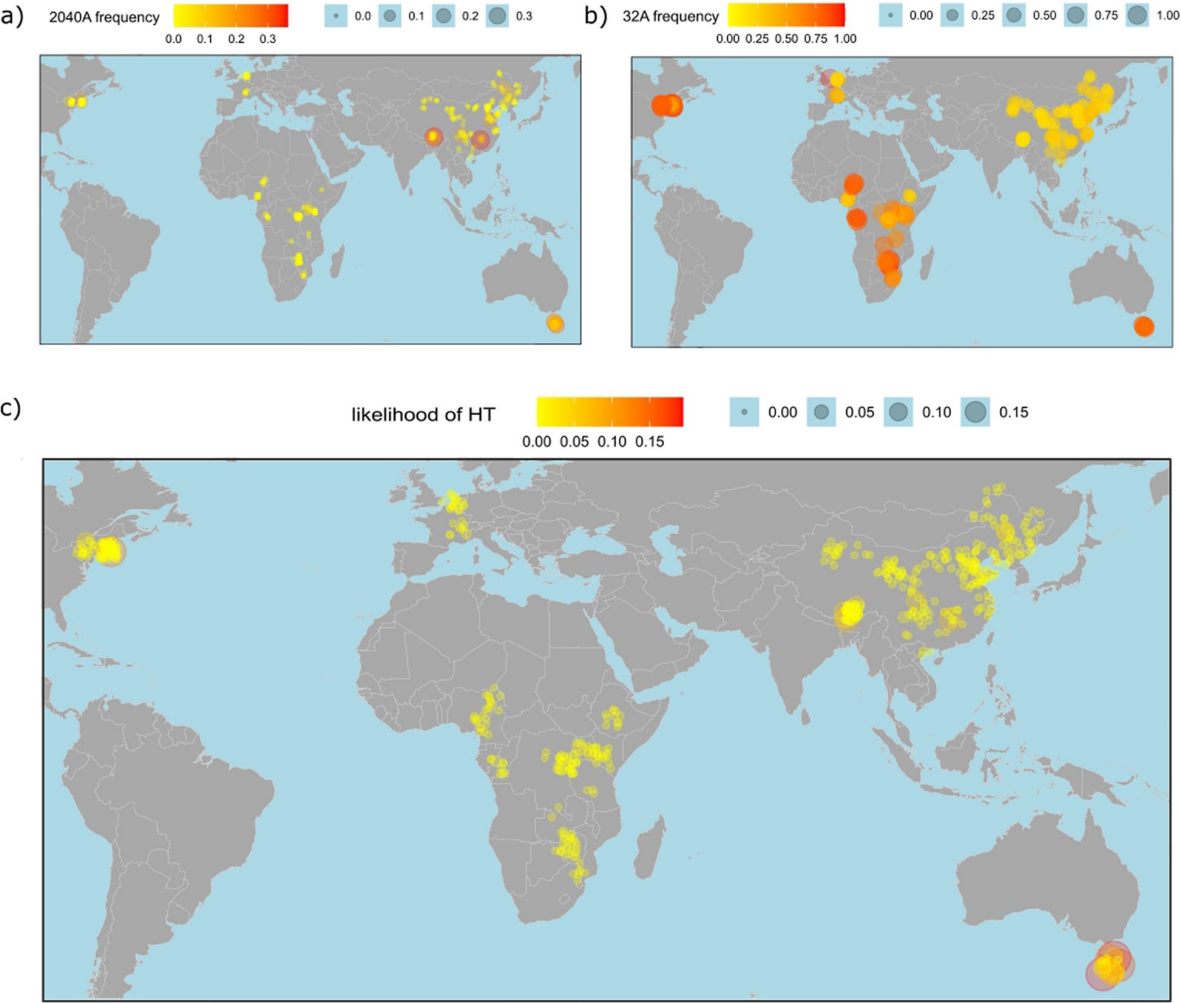
The frequency of the ‘simulans allele’ of *P*-element SNPs segregating in natural *D. melanogaster* populations allows us to identify the geographic region where the HT of the *P*-element from *D. melanogaster* to *D. simulans* most likely occurred. **a** Distribution of 2040A (fixed in *D. simulans*) in natural *D. melanogaster* populations **b** Distribution of 32A (fixed in *D. simulans*) in natural populations of *D. melanogaster* **c** The likelihood that the HT of the *P*-element occurred in a given region can be computed from the frequency of the *D. simulans* allele of all of the five SNPs segregating in *D. melanogaster* populations. Our data suggests that the HT of the *P*-element from *D. melanogaster* to *D. simulans* most likely occurred around Tasmania

We found that 5 SNPs segregate in natural populations of *D. melanogaster* with an average allele frequency difference between *D. melanogaster* and *D. simulans* $>0.01$ (Table 1). We did not find a segregating SNP with a frequency difference $>0.01$ in *D. simulans* populations. The uniformity of the *P*-element in *D. simulans* suggests that a single HT triggered the *P*-element invasion in *D. simulans*, in agreement with previous work [24]. As reported previously the highest difference in allele frequency between *D. simulans* and *D. melanogaster* can be found for the SNP at position 2040 (Table 1). This SNP segregates in *D. melanogaster* populations with the major allele being ‘G’ (99.2%) and the minor allele ‘A’ (0.8%). In *D. simulans* the allele ‘A’ can be found at 100% at this site (Table 1). The frequency of 2040A varies among *D. melanogaster* populations, where the highest frequency can be consistently found in several samples from Tasmania (28%), and isolated samples from China (36%) and Providence (19%; Fig. 3A). The site 2040 is missing in many *P*-element insertions due to a prominent internal deletion (e.g. the *KP*-element [2, 66]). The highest frequency of 2040A may thus not necessarily reflect the highest absolute copy numbers of *P*-element insertions with 2040A. However, also the absolute number of *P*-element insertions having the allele ‘A’ at site 2040 is highest in populations from Tasmania (Supplementary Fig. S6).

Interestingly, we found an additional SNP at position 32, with pronounced variation among *D. melanogaster* populations (Table 1). This SNP has three alleles in *D. melanogaster* (‘T’, ‘A’, ‘G’) and a single one (‘A’) in *D. simulans*. The average frequency of 32A (i.e. the ‘simulans allele’) in *D. melanogaster* is 41%. The highest frequency of 32A can be found in populations from America (94%), Africa (93%) and Tasmania (93%; Fig. 3B). The other three SNPs (at positions 33, 517 and 652) have similar frequencies in natural *D. melanogaster* populations (Supplementary Fig. S7). To infer the most likely origin of the *P*-element HT, it is necessary to integrate the information provided by all five SNPs. To infer a single composite likelihood, we simply computed for each sample $L=\Pi ^{SNP}_i(f^{sim}_i)$ where $f^{sim}_i$ is the frequency of the ‘simulans allele’ in *D. melanogaster* populations for a given SNP *i* (Fig. 3C). The region where *L* is highest is the most likely origin of the *P*-element HT. Based on *L*, we suggest that the HT of the *P*-element from *D. melanogaster* to *D. simulans* most probably happened in or around Tasmania ($L=19\%$) followed by Providence ($L=15\%$) and China ($L=7\%$; Fig. 3C).

To summarize, our data shows that five *P*-element SNPs segregate in *D. melanogaster* populations, whereas we could not find a SNP segregating in *D. simulans* populations. Based on the frequency of the ‘simulans-alleles’ in natural *D. melanogaster* populations, we suggest that the HT of the *P*-element from *D. melanogaster* to *D. simulans* most likely happened in Tasmania or in a neighboring region.

## Discussion

Here, we address the question as to whether the base substitution at site 2040 $G->A$ of the *P*-element was a necessary adaptation that enabled the spread of the *P*-element in natural *D. simulans* populations. Based on the dynamics of different *P*-element versions in experimental populations, we found that that *P*-element without the substitution, i.e. 2040G, is able to spread in *D. simulans*, demonstrating that 2040A is not a necessary adaptation. It is feasible that the *P*-element with 2040G is only able to spread in experimental populations, where rearing conditions are optimized, and that the base substitution 2040 $G->A$ is still necessary for the spread of the *P*-element in natural populations, but we do not consider this scenario likely. Based on experimental data in two species, *D. melanogaster* and *D. simulans*, we further show that the allele at site 2040 (‘G’ or ‘A’) has little impact on the invasion dynamics of the *P*-element. However, one limitation of this work is that the invasion of *P*-element with 2040A failed in *D. simulans* (i.e. in the inbred strain Dsim001) and we resorted to using previously published data of the invasion of 2040A in experimental *D. simulans* populations [25, 26]. This limitation has little impact on our primary conclusion, i.e. that 2040G is able to spread in *D. simulans* populations. Additionally, the finding that the allele at site 2040 (‘G’ or ‘A’) has little impact on the invasion dynamics is supported by data from two species (*D. melanogaster* and *D. simulans*), where experimental conditions were kept absolutely identical in one species (*D. melanogaster*). It is perhaps interesting to ask why the invasion of the *P*-element with 2040A failed in *D. simulans*. In our experience, triggering TE invasions artificially by micro-injection is challenging and frequently fails due to unclear reasons. Possible reasons may be linked to population genetic processes, such as the stochastic early stages of invasions where TEs frequently get lost due to drift [31] or negative selection against *P*-element insertions, and to chromosomal biology (e.g. if the first few insertions end up in silent heterochromatin). It is, however, remarkable that the invasion dynamics of 2040A and 2040G were very similar in *D. simulans*, despite the very different experimental conditions. This could imply that the dynamics of TE invasions are extremely robust to a wide range of different conditions. However, this requires further testing, with experiments where solely one parameter at a time is varied. So far, two factors have been shown to impact the invasion dynamics of the *P*-element. Temperature influences the activity of the *P*-element and thus the speed of the spread, but seems to have little influence on the plateau level [25, 26]. By contrast, in this work we showed that the species influences the plateau level of the invasion, where higher copy numbers were reached in *D. melanogaster* than in *D. simulans*. This could be due to differences in the strength of selection. For example *P*-element insertions could just be more deleterious in *D. simulans* than in *D. melanogaster*. The negative effects of TEs could also arise from ectopic recombination among distant TE insertions, leading to deleterious genomic rearrangements [40, 44, 69]. A higher rate of ectopic recombination in *D. simulans* (e.g. caused by an elevated recombination rate [64]) could thus lead to stronger negative selection against the *P*-element in *D. simulans* than in *D. melanogaster*. An alternative explanation might be that the size of piRNA clusters differs among the species [23]. It is not entirely clear what triggers the host defense against invading TEs in Drosophila [34, 56]. However, the prevailing view, the trap model, posits that an invading TE is silenced when copies of the TE jump into piRNA clusters, which triggers the production of small RNAs that silences the TE [4, 23]. Theoretical works show that under this model the size of piRNA cluster is a major factor determining the number of TEs that accumulate during an invasion [23, 61]. Based on these considerations, the differences in the plateau level among the species suggests that the total size of the piRNA clusters (as a percentage of the genome) might be larger in *D. simulans* than in *D. melanogaster*. It is interesting that the overall TE content seems to be lower in *D. simulans* than in *D. melanogaster* [62, 63]. It is thus feasible that the forces leading to the accumulation of fewer *P*-element insertions in *D. simulans* are generally driving the lower TE content of *D. simulans* as compared to *D. melanogaster*.

The *P*-element invasion in natural *D. simulans* populations lags behind the invasion of *D. melanogaster* populations by about 30–50 years (*D. melanogaster*: 1950–1980; *D. simulans*: 2006–2014 [1, 19, 22, 24]). In a previous work, we speculated that this delay could be due to the waiting time for a necessary mutation, 2040G$->$ 2040A, that enabled the spread of the *P*-element in *D. simulans* [24]. Our data however suggest that this base substitution was not necessary, as the *P*-element with 2040G is able to rapidly spread in experimental *D. simulans* populations. The reason for the $\approx$30 years lag between the invasions in the two species is likely the rarity of HT events. In agreement with a single HT triggering the invasion, the absence of segregating *P*-element SNPs in *D. simulans*, especially of the SNPs segregating at a high frequency in *D. melanogaster*, suggests that the *P*-element invasion in *D. simulans* was triggered by a single HT event (see also Kofler et al. [24]). Assuming that Drosophila species have about 15 generations per year [46], it took the *P*-element between 450–750 generations before it spread in *D. simulans*. This high number of generations is especially remarkable, given the large population size and cosmopolitan distribution of both species [8]. Given the plentiful opportunities for HT, it seems that a successful HT (i.e. a HT that triggers an invasion) is exceedingly rare, and it will be challenging to identify the elusive vectors for HT. Many possible vectors have been proposed, such as mites, viruses and Wolbachia (intracellular bacteria of certain insects) [16, 20, 33]. Due to this rarity, it may be nearly impossible to identify direct evidence for the vector being responsible for triggering an invasion. One alternative strategy may be to indirectly narrow in on the possible vectors of HT, e.g. by finding vectors that carry TE fragments [16, 20]. The identification of the geographic region where a HT happened could also help to narrow in on potential vectors of the HT and perhaps on environmental conditions that are beneficial for successful HT (e.g. temperatures that increase the activity of a TE could facilitate HT). For the HT of the *P*-element from *D. willistoni* to *D. melanogaster* the geographic origin can be inferred from the habitat of the involved species (likely South or Central America, the habitat of *D. willistoni*). This strategy cannot be used for *D. melanogaster* and *D. simulans*, as both species are largely cosmopolitan [8]. We thus reasoned that the most likely geographic origin is the region where *D. simulans* alleles have the highest frequency in natural *D. melanogaster* populations. Based on this analysis we suggest that Tasmania, or a neighboring region, is the most probable origin of the HT. In Tasmania, several samples have a high likelihood of the HT, whereas only a single sample has a high likelihood in Providence (USA) and China. One limitation of our approach is that data are missing for some geographic regions. Although we analyzed 547 samples from 5 continents [10, 17, 28, 47, 48, 54], we lack samples from South America, Northern Africa, Central Asia and Mainland Australia. It is thus possible that the likelihood for HT is even higher in any of these aforementioned regions than in Tasmania. Community efforts, such as DrosEU [21], may provide more dense sampling of global *D. melanogaster* strains and thus help to refine the geographic origin of the HT that triggered the *P*-element invasion in *D. simulans*.

## Methods

### Site-directed mutagenesis

For *P*-elements with 2040G, we relied on the plasmid ppi25.1 (kindly provided by Dr. Erin Kelleher). To obtain a *P*-element with 2040A we altered ppi25.1 using site directed mutagenesis with the Q5 Site-Directed Mutagenesis Kit E0552S (New England Biolabs, Ipswich, MA, USA) following manufacturer’s protocol with the primers (forward: GAAAGTTTCAATTGAGAATGTAG; reverse: CTGAAACATATAGCTAAACATTAAAC).

### Transformation and experimental populations

The ppi25.1 plasmids with 2040A and 2040G were injected into embryos of the strains ‘Dmel68’ (collected 1954 in Israel; *D. melanogaster*) and ‘Dsim001’ (collected 1956 in Geoergetown Guyana; *D. simulans*) by Rainbow Transgenic Flies, Inc. (Camarillo, CA, USA). Injected females were separated individually and then allowed to lay eggs. We established lines from the offspring and confirmed the presence of the *P*-element using PCR. To set up the experimental populations, we mixed 50 pre-mated flies (25 flies from the transformed lines mated with 25 naive flies) with 200 naive flies (total population size $N=250$). The populations were maintained at 25$^{\circ }$C with non-overlapping generations.

### Data analysis

To estimate TE copy numbers in the samples, we used our tool DeviaTE (v0.3.8) [65]. We trimmed all reads to a length of 100bp and aligned the reads to a set of sequences consisting of the *P*-element (from *D. melanogaster*) and three single-copy-genes (SCGs) (*rhi*, *tj*, *RpL32*). We used the corresponding orthologs of these single copy genes in *D. melanogaster* and *D. simulans*. Finally, we estimated the copy number of the TE with DeviaTE, which estimates the copy numbers of a TE by normalizing the coverage of the TE to the coverage of the SCGs. DeviaTE also provides the count of each nucleotide at each site of the *P*-element which allows us to compute the allele frequency of *P*-element SNPs. To identify species specific SNPs, we obtained, for each species, short-read data from three recently sequenced strains (*D. melanogaster*: Iso-1, Oregon-R, Canton-S; *D. simulans*: Mod6, w501, wXD1 [11, 37]) and then used DeviaTE. The species-specific SNPs were identified with a custom Python script (diagnosticSNPs-finder.py) The full list of species-specific SNPs in the three SCGs can be found in Supplementary data S1, S2.

### Natural populations

To infer the frequency of *P*-element SNPs in natural populations, we gathered publicly available data for *D. melanogaster* ([10, 17, 28, 47, 48, 54]) and *D. simulans* ([24, 55, 57, 58, 64]). Copy numbers of the *P*-element and the allele frequencies of the SNPs were estimated with DeviaTE as described earlier. Subsequent data analysis has been conducted in R. For an overview of all data from natural populations used in this work, see Supplementary data S3, S4.

## Supplementary Information

Supplementary Material 1.

## Data Availability

The sequencing data generated in this work are available from NCBI with the primary accession code PRJNA997230. Analyses of this work were documented with R Markdown and made available at GitHub (https://github.com/filwierz/2040A_P-element).
