# Supplementary material for: On the origin of the P-element invasion in Drosophila simulans
Source: Mob DNA. 2025 Feb 26;16:7. doi: 10.1186/s13100-025-00345-0 (PMC11863927; doi:10.1186/s13100-025-00345-0)
Supplement: Supplementary file 1 — Supplementary Material 1. [file 13100_2025_345_MOESM1_ESM.zip › supplement.pdf]

Supplementary figures and tables

January 22, 2025

Supplementary figures

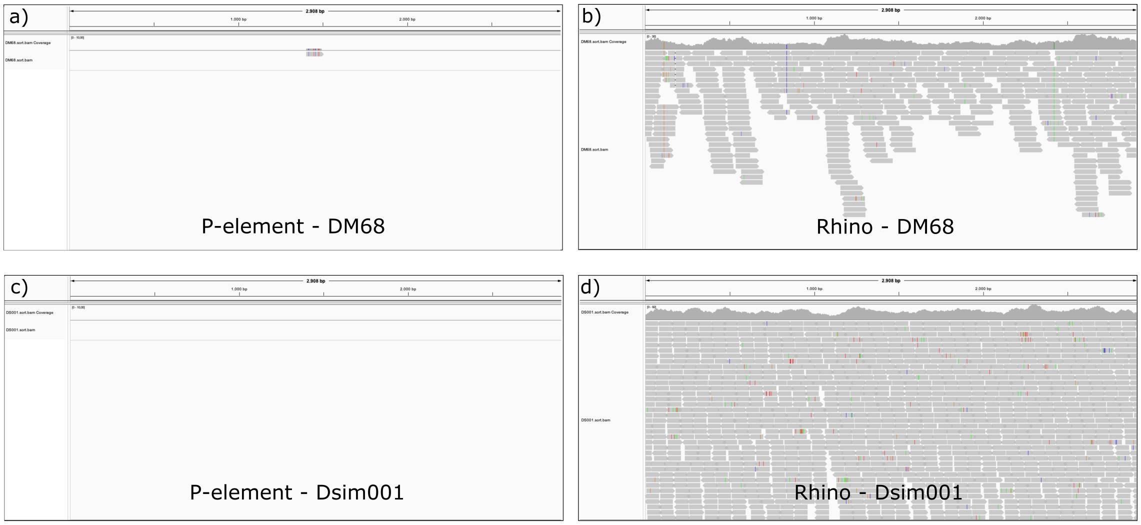

Figure S1: The P-element is absent in the *D. melanogaster* strain Dmel68 (DM68) and the *D. simulans* strain Dsim001. Short-read data from both strains were aligned to the *P*-element and the single copy gene *rhi* (Rhino; the correct ortholog was used in each species). Plots show IGV screenshots of the reads mapping to the *P*-element (a,c) and Rhino (b,d) in *D. melanogaster* (a,b) and *D. simulans* (c,d).

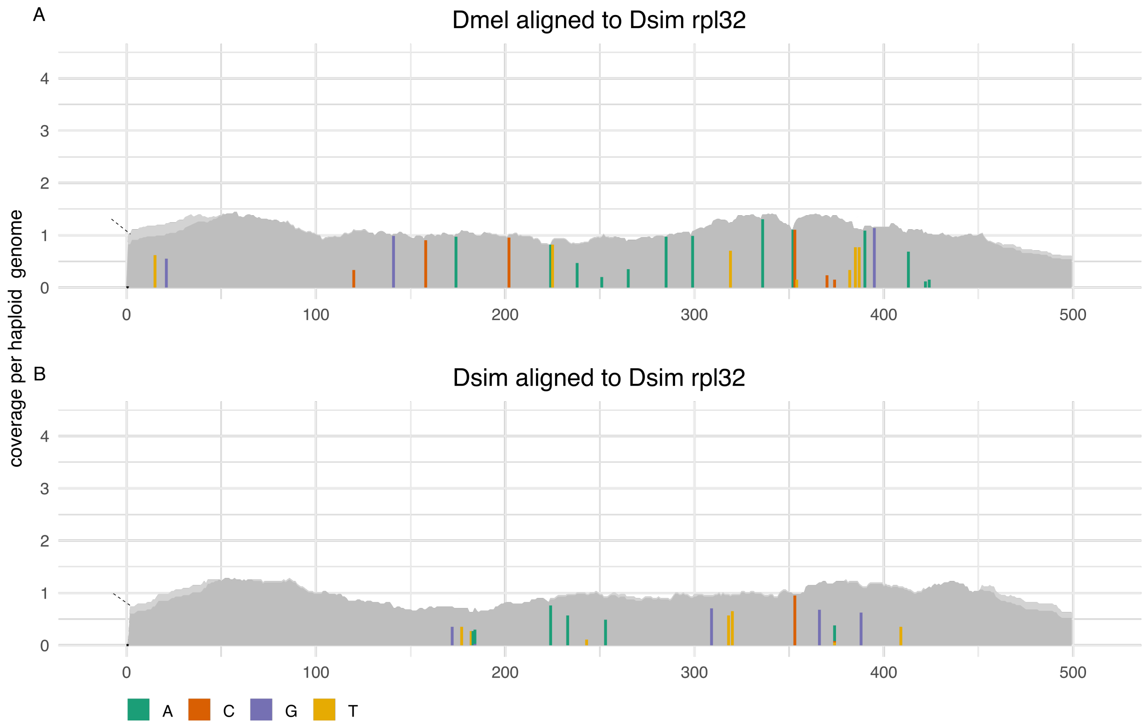

Figure S2: Diagnostic SNPs enable us to identify contamination of data with reads from a different species. DeviaTE plots show the coverage and the frequency of SNPs (colored bar) for *D. melanogaster* and *D. simulans* reads aligned to the *D. simulans* ortholog of *RpL32*. *D. melanogaster* contamination of *D. simulans* data can be identified based on SNPs that are fixed for alternative alleles in the two species.

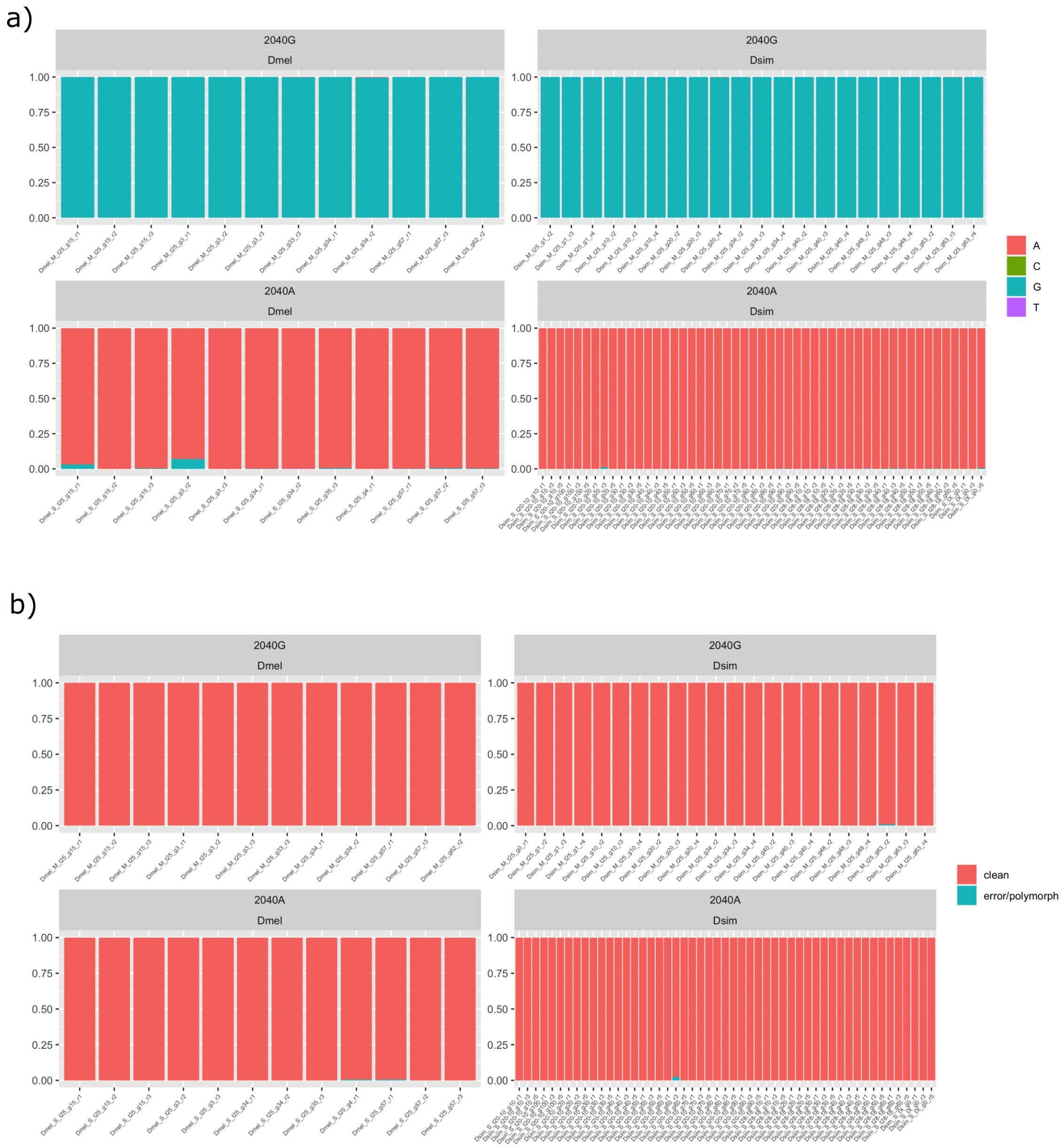

Figure S3: Diagnostic SNPs confirm that all data from the experimental populations correspond to the correct allele of the *P*-element (A) and the correct species (B). A) Frequency of alleles at site 2040 in the different data sets. B) Frequency of species-specific SNPs in the different data sets. The average frequency of the allele from the correct species is shown as red bar, whereas the frequency of the allele from the other species is shown as green bar.

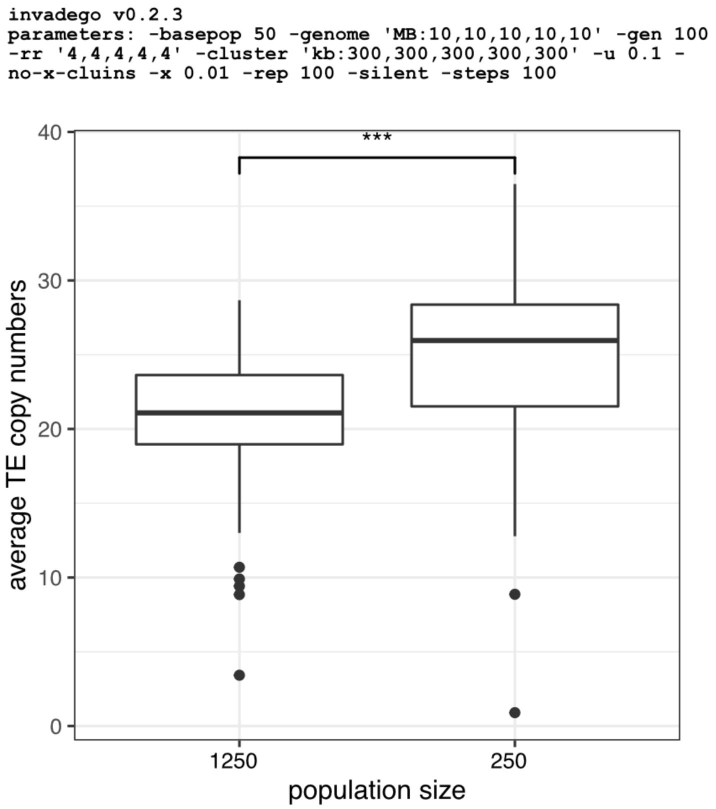

Figure S4: Our simulations show that TE invasions in small population size will result in higher TE load than invasions in larger populations. This makes intuitive sense as the efficacy of selection ($Ne * x$, where x is the negative effect of a TE insertion) is higher in large populations. Differences in the population size between 2040G ($N = 250$) and 2040A ($N = 1000$) in *D. simulans* can thus likely not explain the slightly lower plateau level of 2040G as compared to 2040A (the small population has fewer TEs and not more as expected). We performed 100 simulations of TE invasions with two different population sizes using our tool InvadeGO [Scarpa and Kofler, 2023]. Simulations with piRNA clusters accounting for 3% of the genome were performed and the average TE copy numbers at generation 100 are shown. The exact parameters are provided on top of the graph.

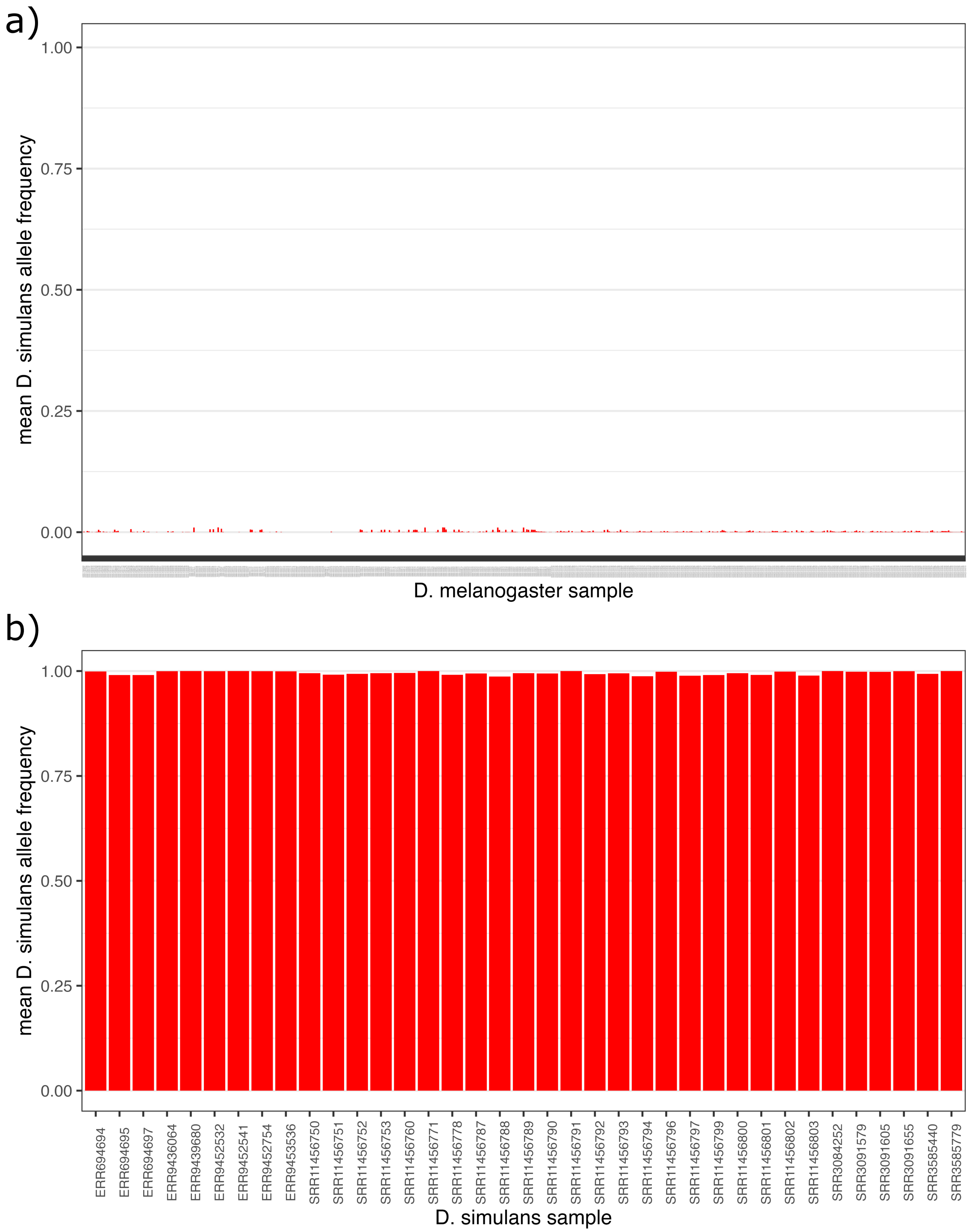

Figure S5: Diagnostic SNPs confirm that all publicly available data from natural populations of *D. melanogaster* (A) and *D. simulans* (B) are largely free of contamination from the other species. Reads were aligned to *D. melanogaster* single-copy-genes and the average frequency of the *D. simulans* allele was estimated (red bar).

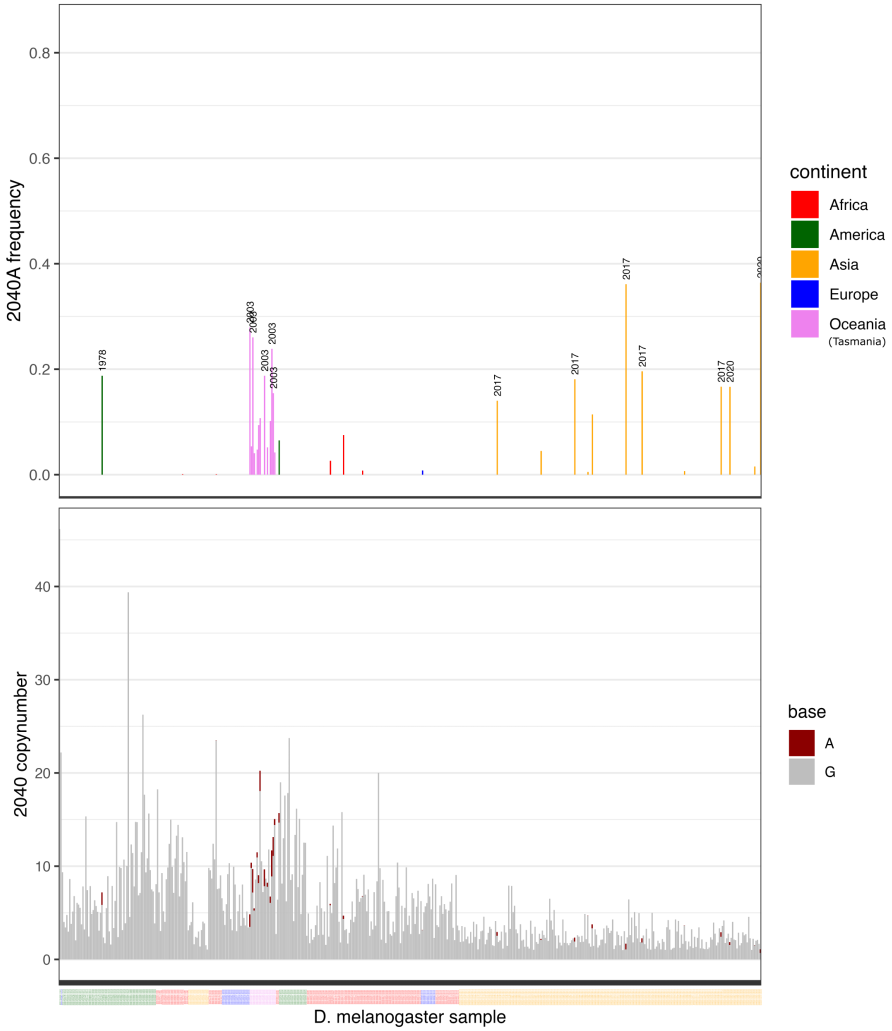

Figure S6: Frequency and copy numbers of P-element insertions with 2040A in *D. melanogaster* populations from 5 different continents. Numbers on top refer to sampling year of the strain (or population). Note that due to the presence of P-element insertions with internal deletions the copy number estimates of insertions having the site 2040 may be quite low in some regions.

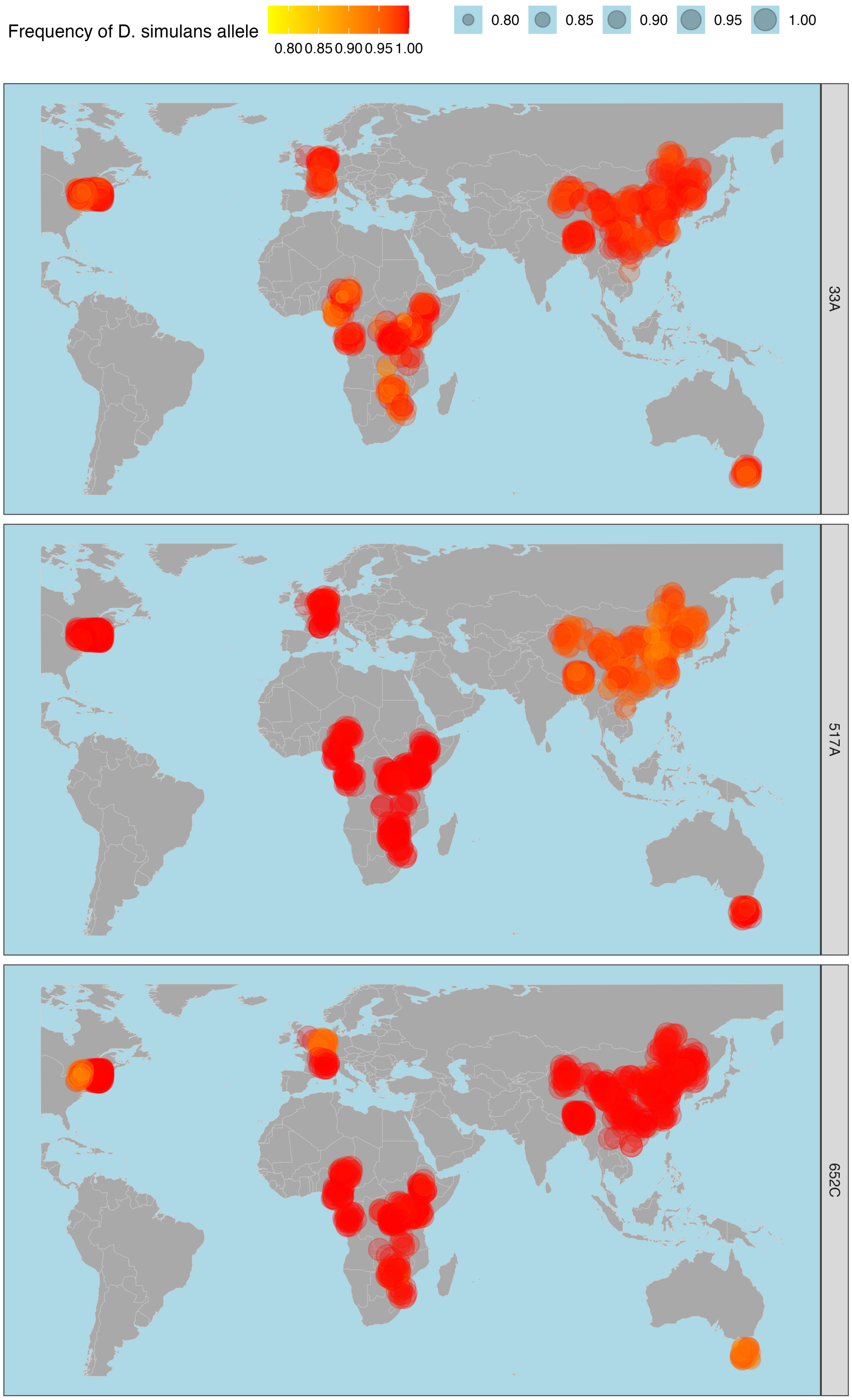

Figure S7: Frequency of the 'simulans-allele' in natural *D. melanogaster* populations. Data are shown for three different SNPs - at positions 33, 517 and 625 - of the *P*-element

References

A. Scarpa and R. Kofler. The impact of paramutations on the invasion dynamics of transposable elements. *Genetics*, 225 (4):iyad181, 2023.
